# Supplementary material for: Barriers to the Provision of Preventive Care to People Living with Mental Health Conditions: Self-Report by Staff Working in an Australian Community Managed Organisation
Source: Int J Environ Res Public Health. 2022 Apr 7;19(8):4458. doi: 10.3390/ijerph19084458 (PMC9027436; doi:10.3390/ijerph19084458)
Supplement: Supplementary file 1 [file ijerph-19-04458-s001.zip › ijerph-1648641-supplementary.pdf]

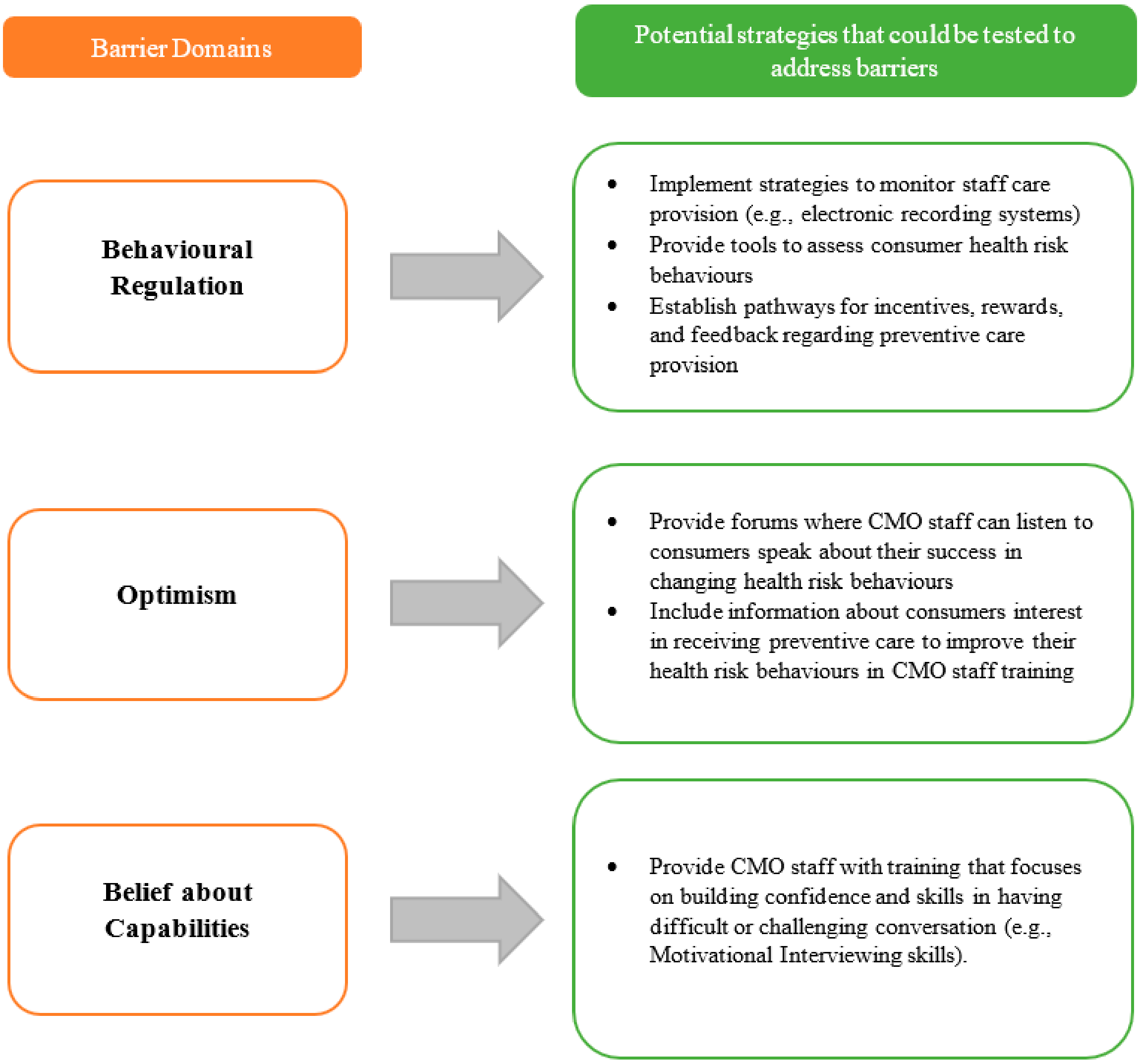

Figure S1. Top Three Barrier Domains reported by CMO staff and potential strategies that could be tested to address these barriers.

Table S1. All TDF survey items and responses.

		Strongly Disagree	Disagree	Somewhat Disagree	Unsure	Somewhat Agree	Agree	Strongly Agree
		%	%	%	%	%	%	%
**Knowledge and Skills**								
	I have the knowledge and skills to ask people accessing the service:							
1	Whether they smoke (n=185)	0.5	0.5	1.6	2.2	8.6	31	55
2	About their fruit and vegetable consumption (n=186)	0	0.5	1.1	4.8	7.5	36	51
3	How many standard drinks of alcohol they consume (n=185)	0	2.2	1.1	6.5	11	33	46
4	About their current physical activity (n=186)	0	0.5	1.1	3.2	7.0	36	52
	I have the knowledge and skills to provide brief advice to people accessing the service on how to:							
5	Quit or reduce smoking (n=185)	0.5	2.7	1.1	5.4	17	33	41
6	Increase their fruit and vegetable consumption (n=186)	0	1.1	2.7	3.8	8.6	40	44
7	Reduce their alcohol consumption (n=185)	0	2.7	3.2	6.5	15	34	40
8	Increase their physical activity (n=186)	0	1.1	2.2	3.2	7.0	40	46
	I have the knowledge and skills to connect people accessing the service with behaviour change support services to help them with:							
9	Quitting smoking or reducing the amount they smoke (n=184)	0.5	2.2	2.7	5.4	13	36	40
10	Increasing their fruit and vegetable consumption (n=185)	0.5	1.6	1.6	4.3	8.6	41	43
11	Reducing their alcohol consumption (n=185)	0.5	1.1	2.7	5.9	12	40	38
12	Increasing their physical activity (n=185)	0	0.5	1.6	3.8	12	39	43
	I know where I can connect people so they can get help with:							

13	Quitting smoking or reducing the amount they smoke (n=182)	0	2.7	1.6	2.7	18	35	41
14	Increasing their fruit and vegetable consumption (n=183)	0	1.6	1.6	3.3	13	40	41
15	Reducing their alcohol consumption (n=183)	0	1.6	1.6	4.4	16	35	42
16	Increasing their physical activity (n=183)	0	2.2	0.5	2.2	12	40	44
**Social and Professional Role and Identity**								
17	[a] I believe providing preventive support is as important as other aspects of my role (n=177)	1.1	5.6	7.3	6.2	25	39	15
18	I believe it should be part of my role to provide preventive support to people accessing the service (n=168)	0	4.8	0.6	3.0	21	41	30
19	I believe it should be part of my organisation's role to provide preventive support (n=165)	0	0.6	2.4	8.5	17	41	31
**Belief About Capabilities**								
	I am confident that I can provide preventive support to people accessing the service regarding:							
20	Quitting smoking or reducing the amount they smoke (n=181)	0.6	2.2	2.2	6.1	19	43	27
21	Increasing their fruit and vegetable consumption (n=182)	0.5	1.6	1.6	4.4	12	46	34
22	Reducing their alcohol consumption (n=182)	0.5	1.6	3.3	6.0	18	42	28
23	Increasing their physical activity (n=182)	0.5	1.6	1.1	4.4	12	42	38
	[a] I do not find it challenging to provide preventive support to people accessing the service regarding:							

24	Quitting smoking or reducing the amount they smoke (n=181)	8.3	20	23	6.1	8.8	20	13
25	Increasing their fruit and vegetable consumption (n=182)	6.0	18	20	6.6	15	20	14
26	Reducing their alcohol consumption (n=182)	6.0	20	21	8.2	10	21	13
27	Increasing their physical activity (n=182)	8.8	16	19	6.0	13	21	16
**Optimism**								
28	[a] I am optimistic that people accessing the service will follow-up the connections I make to health behaviour supports (n=178)	3.4	16	25	20	17	15	3.9
29	I am optimistic that my organisation could provide effective preventive support (n=178)	0	0	1.7	7.3	14	43	35
**Belief About Consequences**								
30	[a] I believe providing preventive support will not have a negative impact on the relationship I have with people accessing the service (n=180)	2.2	5.6	8.3	13	17	36	18
31	I believe providing preventive support to people accessing the service will benefit their physical health (n=176)	0.6	0.6	0.6	2.3	9.7	42	44
32	I believe providing preventive support to people accessing the service will benefit their mental health (n=176)	1.7	0.6	0.6	2.9	6.9	36	51
**Reinforcement**								
33	People accessing the service appreciate the preventive support I offer them (n=157) [b]	1.3	1.9	2.5	23	31	30	10
34	I receive a sense of satisfaction when I provide preventive support (n=151) [b]	0.7	0	1.5	8.8	20	37	32
35	[a] I receive recognition from my employer when I provide preventive support (n=141) [b]	4.3	4.3	14	26	11	27	14

**Intentions**								
36	I would like to improve my provision of preventive support (n=173)	0	3.5	1.2	6.4	20	44	25
**Goals**								
37	It is a reasonable goal to provide preventive support to all people accessing the service (n=166)	0	4.8	7.8	11	15	36	26
**Memory, Attention, and Decision Processes**								
38	[a] Providing preventive support is not something I forget to do (n=165)	5.5	4.8	18	14	18	35	4.8
**Environmental Context and Resources**								
39	[a] Providing preventive support to people accessing the service is feasible (n=177)	0.6	3.4	2.8	8.5	20	41	23
40	Preventive support should be offered to people accessing the service whether or not they have expressed an interest (n=178)	1.1	2.8	4.5	14	22	32	24
41	[a] A person's interest does not drive whether I provide them with preventive support (n=175)	13	26	30	11	8.6	9.7	1.1
42	[a] Improving the physical health of individuals with a mental health issue is a priority within the mental health sector (n=172)	1.2	3.5	9.9	5.8	18	36	26
43	[a] Preventive support fits well with a person-led approach (n=166)	0.6	2.4	13	16	17	37	15
44	Preventive support fits well with my general provision of support (n=167)	0	2.4	0.6	15	23	40	20
45	I would welcome more resources and aids to assist my provision of preventive support (n=167)	0.6	0	0	6.6	17	35	41
46	There is someone within my organisation who can provide advice or guidance about my provision of preventive support (n=164)	0	3.0	6.1	26	14	36	15

47	[a] There is enough time to provide preventive support to people accessing the service (n=165)	5.5	4.8	18	14	18	35	4.8
48	[a] It is possible to tailor preventive support to the individual needs of people accessing the service (n=166)	0	2.4	5.4	14	22	39	18
49	[a] My manager expects me to provide preventive support (n=177)	1.1	5.6	5.6	14	14	40	20
50	My manager encourages me to provide preventive support (n=176)	1.1	8.5	4.0	12	17	33	24
51	My manager encourages me to take part in training in the delivery of preventive support (n=174)	2.9	14	5.2	18	14	25	20
52	My manager provides feedback on my provision of preventive support (n=150) [b]	6.0	13	6.0	17	19	29	10
53	My organisation monitors the provision of preventive support (n=173)	0	6.9	5.2	30	18	32	8.1
54	My organisation provides guidelines on the delivery of preventive support (n=174)	0	6.3	5.2	28	20	32	8.0
55	[a] My organisation has provided me with training in the provision of preventive support (n=172)	8.1	26	24	8.7	13	16	4.1
56	My organisation is committed to improving the physical health of individuals experiencing a mental health issue (n=175)	0	0.6	0.6	7.4	9.7	42	40
57	My organisation provides resources and aids for me to use in prompting my provision of preventive support (n=168)	0	8.3	9.5	23	19	29	11
58	My organisation provides resources and aids for me to use in recording a person's health risk behaviours (n=168)	0.6	4.2	3.6	14	18	42	18

59	My organisation provides resources and aids for me to use in delivering behaviour change advice (n=167)	0.6	5.4	6.0	22	19	39	8.4
60	My organisation provides resources and aids for me to use in connecting people to behaviour change services (n=167)	0.6	4.2	4.8	18	20	40	13
61	My organisation provides resources and aids for me to use in recording my provision of preventive support (n=168)	1.2	6.0	6.5	24	18	35	10
**Social Influence**								
62	I believe people accessing the service would expect me to provide them with preventive support (n=174)	0.6	4.6	8.6	13	22	37	14
63	[a] I believe people accessing the service would find it acceptable for me to provide them with preventive support (n=172)	0	1.2	5.2	12	20	42	19
64	I believe other staff in my organisation think I should deliver preventive support (n=166)	1.2	8.4	3.0	42	15	26	5.4
**Emotion**								
65	[a] I do not feel (or would not feel) burdened by the additional responsibility of providing preventive support (n=174)	2.3	2.9	12	9.2	21	37	16
66	[a] I do not feel (or would not feel) hypocritical when providing preventive support (n=167)	0	3.6	17	11	16	38	14
67	[a] I do not feel (or would not feel) uncomfortable when providing preventive support (n=167)	1.2	3.0	13	13	18	42	11
**Behavioural Regulation**								
68	I monitor the preventive support I provide to people accessing the service (n=179)	1.1	7.3	3.9	11	21	41	16
69	[a] Providing preventive support is something I do routinely (n=177)	4.5	14	19	4.5	16	32	9.6

[a] This item was originally negatively worded, wording and scoring has been reversed in the table

[b] This items was only answered by participants who reported providing preventive care (participants could choose a 'not applicable' response option if they did not provide preventive care).
